# Ten‐Year Outcome of a Patient With Concurrent Pelvic Myeloid Sarcoma and Underlying Chronic Myeloid Leukaemia in Chronic Phase: A Case Report and Literature Review

**DOI:** 10.1002/cnr2.70514

**Published:** 2026-03-12

**Authors:** Kar Ying Yong, Soo How Lim, Lee Gong Lau, Lee Ping Chew

**Affiliations:** ^1^ Haematology Unit, Medical Department Miri General Hospital Miri Sarawak Malaysia; ^2^ Department of Clinical Research Centre Miri General Hospital Miri Sarawak Malaysia; ^3^ Institute for Clinical Research, National Institute of Health Selangor Malaysia; ^4^ Borneo Medical Centre Kuching Sarawak Malaysia; ^5^ Department of Haematology Sarawak General Hospital Kuching Sarawak Malaysia

**Keywords:** case report, chronic myeloid leukaemia, haematology, myeloid sarcoma, oncology, radiotherapy, tyrosine kinase inhibitor

## Abstract

**Background:**

Myeloid sarcoma is a rare solid tumour of immature myeloid precursors occurring in an extramedullary site. It often presents significant diagnostic and therapeutic challenges to clinical haematologists.

**Case:**

We describe a previously healthy patient who presented simultaneously with hyperleucocytic chronic myeloid leukaemia in chronic phase (CML‐CP) and a pelvic myeloid sarcoma (MS). She failed to respond to conventional intensive chemotherapy meant for acute myeloid leukaemia but responded well to a combination of radiotherapy and tyrosine kinase inhibitor. She has remained well and in deep molecular response for the past 10 years.

**Conclusion:**

We report the first case of MS in CML‐CP that has achieved 10 years of long‐term remission with a combination of radiotherapy and tyrosine kinase inhibitor, without allogeneic stem cell transplantation. We also reviewed the literature on CML‐CP with simultaneous MS to determine the disease characteristics and treatment outcomes.

## 1 Introduction

Myeloid sarcoma (MS) is a rare extramedullary tumour composed of immature myeloid precursor cells occurring at site other than the bone marrow. It is also known as granulocytic sarcoma or chloroma due to the greenish‐yellow appearance. The most common sites described include skin, soft tissue, bone, and lymph node [1, 2]. MS is commonly associated with acute myeloid leukaemia (AML), myeloproliferative neoplasm (MPN), and myelodysplastic syndrome (MDS). The condition may precede, coincide with or manifest as a disease relapse of any of the aforementioned myeloid neoplasms. However, because of its rarity, MS often poses diagnostic and therapeutic challenges clinically, especially if it occurs in association with a myeloid neoplasm other than AML. MS occurs in 12 to 20% of CML cases [3]. Here, we report a middle‐aged woman who presented simultaneously with hyperleucocytic chronic myeloid leukaemia in chronic phase (CML‐CP) and a pelvic MS. The initial response to conventional AML‐type chemotherapy was poor. However, treatment with a combination of radiotherapy and tyrosine kinase inhibitor (TKI) without allogeneic hematopoietic stem cell transplantation (HSCT) resulted in remarkable therapeutic success. We also conducted a literature review, aiming to explore the association of MS with CML‐CP and to evaluate the efficacy of various potential treatment strategies. We identified a total of 27 reported cases of MS occurring in patients with CML‐CP that were published between 1989 and 2022. We noted that there were heterogeneous treatment strategies and outcomes in managing these patients. We highlight the important role of TKI in controlling the systemic disease, while radiotherapy and surgery seem to be able to provide good local disease control in selected cases. Our case appears to be the first reported case of MS in CML‐CP achieving a durable response up to 10 years with just a combination of radiotherapy and TKI.

## 2 Methods

A comprehensive literature search was conducted using the PubMed/MEDLINE database to identify relevant articles published between January 1980 and November 2025. PubMed/MEDLINE was selected as it provides extensive coverage of peer‐reviewed biomedical and clinical research and is considered the most appropriate primary database for rare haematologic malignancies. To ensure high sensitivity and reproducibility, the search strategy utilized a combination of Medical Subject Headings (MeSH) and free‐text keywords relevant to myeloid sarcoma and chronic myeloid leukaemia. The exact Boolean search employed was: ((“Sarcoma, Myeloid” [Mesh] OR “Granulocytic Sarcoma” [Title/Abstract] OR “Myeloid Sarcoma” [Title/Abstract] OR Chloroma [Title/Abstract]) AND (“Leukaemia, Myelogenous, Chronic, BCR‐ABL Positive” [Mesh] OR “Chronic Myeloid Leukaemia” [Title/Abstract] OR CML[Title/Abstract])).

We screened the resulting citations to identify studies reporting on patients with Myeloid Sarcoma (MS) specifically occurring during the chronic phase of Chronic Myeloid Leukaemia (CML‐CP). From the selected articles, we extracted and analyzed demographic and clinical data, including age, sex, CML phase at the time of MS diagnosis, anatomical site of involvement, treatment modalities, response to therapy, and clinical outcomes. The primary objective of this review was to evaluate the various management strategies and to determine the most effective therapeutic approach for MS in the setting of CML‐CP.

Inclusion criteria:

1. Patients above 12 years old with either newly diagnosed or pre‐existing CML‐CP who developed MS during the course of their disease
2. Only articles published in English were included.

Exclusion Criteria:

1. CML in accelerated phase (CML‐AP)
2. CML in blast crisis (CML‐BC)
3. MS occurring after allogenic stem cell transplantation
4. Studies lacking sufficient biological and treatment data
5. Studies in language other than English.

## 3 Case Report

**FIGURE 1:**
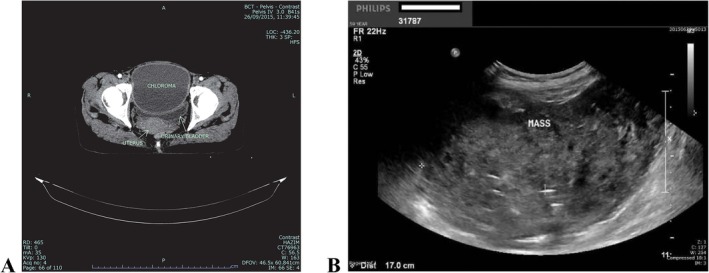
(A) CT and (B) ultrasound scan pictures of the pelvic mass.

A 60‐year‐old woman, with no significant past medical history, presented to Sarawak General Hospital, Kuching, in June 2015 with a two‐month history of abdominal distension, exertional dyspnoea, anorexia, and weight loss. There were no fever as well as gastrointestinal, urinary, or gynaecological symptoms. On physical examination, a large pelvic mass was palpated, extending up to the level of the umbilicus, which was initially thought to be a pelvic malignancy. Ultrasonography (Figure 1B) and computed tomography (CT) of the abdomen (Figure 1A) revealed a 12 cm × 10 cm × 12 cm solid mass in the pelvis anterior to the urinary bladder and uterus, in contact with the anterior abdominal wall and the adjacent bowel loops. Splenomegaly was also noted, with a splenic span of 17 cm.

**FIGURE 2:**
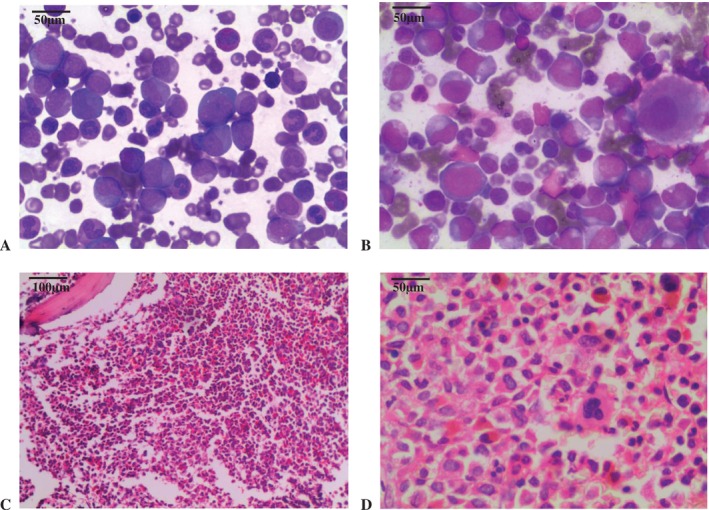
(A) Peripheral blood film showing hyperleukocytosis and myeloid cells at varying stages of maturation (×400). (B) Bone marrow smear showing a similar pattern to peripheral blood (×400). (C) and (D) Bone marrow trephine biopsy on H&E showing marked hypercellularity due to myeloid proliferation (×100 and ×400, respectively).

Complete blood count (CBC) showed hyperleukocytosis with white cell count of 362 × 10^9^/L (normal: 4–11 × 10^9^/L), anaemia with haemoglobin level of 5·5 g/dL (normal: 11.5–15.2 g/dL), and thrombocytosis with platelet count of 643 × 10^9^/L (normal: 150–400 × 10^9^/L). Peripheral blood film demonstrated marked leukocytosis with myeloid cells at varying stages of maturation (Figure 2A). A subsequent bone marrow aspirate revealed markedly hypercellular marrow with myeloid predominance (myeloid to erythroid ratio of 22:1) and 1% blasts suggestive of chronic myeloid leukaemia in chronic phase (CML‐CP), with findings consistent with the bone marrow smear and trephine biopsy (Figure 2B–D). An ultrasound‐guided percutaneous trucut biopsy of the pelvic mass revealed diffuse infiltration by immature cells positive for CD45 and myeloperoxidase, consistent with the diagnosis of myeloid sarcoma. Cytogenetic analysis of the bone marrow showed the presence of the Philadelphia chromosome, and molecular study detected the BCR‐ABL fusion transcript (e13a2 subtype), confirming a diagnosis of chronic myeloid leukaemia (CML) in chronic phase.

The patient was initially treated with hydroxyurea for cytoreduction while waiting for the cytogenetic and genetic results. Although the hyperleukocytosis resolved gradually, the pelvic mass remained unchanged. Upon confirmation of the diagnosis of myeloid sarcoma (MS), the patient was administered AML‐type induction chemotherapy in combination with imatinib daily. We induced with MA (3 + 5), consisting of mitoxantrone 8 mg/m^2^/day, for 3 days and cytarabine 100 mg/m^2^/day, for 5 days, followed by MiDAC, consisting of mitoxantrone (12 mg/m^2^) and cytarabine (1500 mg/m^2^). Despite the aggressive treatment, the size of the pelvic mass did not significantly reduce, and the patient experienced marked myelosuppression and pancytopenia post‐chemotherapy. Given the poor response to chemotherapy, we decided to give radiotherapy to the pelvic mass while continuing to treat the systemic disease with imatinib. Radiotherapy was delivered to a total dose of 36 Gy over 20 fractions, followed by an additional boost dose of 9 Gy delivered over five fractions. This combined approach led to the eventual complete resolution of the pelvic MS. A follow‐up bone marrow examination confirmed both morphological and complete cytogenetic remission. The patient was maintained on imatinib 400 mg daily, and she achieved major molecular response with 4·5‐log reduction of BCR‐ABL fusion transcripts since 2018 till today. We last saw her in May 2025, and she had remained clinically well and in complete molecular remission without the need for further chemotherapy. The patient's clinical course, from initial presentation to follow‐up, is summarized in Table 1.

**TABLE 1:** Timeline of events from the day of presentation to follow‐up.

Day 0	Presented with abdominal distension, exertional dyspnoea, and constitutional symptoms Noted a large pelvic mass and splenomegaly clinically; confirmed by ultrasound examination Complete blood count showed hyperleukocytosis (WCC 362 × 10^9^/L), anaemia (Hb 5.5 g/dL), and thrombocytosis (platelets 643 × 10^9^/L) Peripheral blood film was suggestive of CML‐CP
Day 1	Bone marrow examination demonstrated hypercellular marrow with myeloid predominance and 1% blasts, suggestive of CML‐CP Started hydroxyurea as a cytoreductive agent
Day 3	Ultrasound guided biopsy of pelvic mass
Day 10	Histology confirmed the diagnosis of MS
Day 14	Cytogenetics and molecular tests confirmed the diagnosis of CML in CP
Day 15	Started chemotherapy with MA (3 + 5) and oral imatinib No resolution of the pelvic mass size
Day 45	Started chemotherapy, MIDAC, and oral imatinib No resolution of the pelvic mass size
Day 90	Radiotherapy to the pelvic mass together with oral imatinib
4th month	Complete resolution of the pelvic mass Achieved major molecular response (BCR‐ABL titre by PCR method)
6 month up to 10 years	Remained in major molecular response (BCR‐ABL titre by PCR method) Patient compliant to imatinib

Abbreviations: CML‐CP, chronic myeloid leukaemia in chronic phase; Hb, haemoglobin; MA, mitoxantrone and arac‐C; MiDAC, mitoxantrone and ara‐C; MS, myeloid sarcoma; PCR, polymerase chain reaction; WCC, white cell count.

## 4 Results

We reviewed 14 articles, published between 1989 and 2022, that described a total of 27 patients with CML‐CP and MS who were managed with various treatment strategies, including TKI, surgical intervention, radiotherapy, systemic chemotherapy, and hydroxyurea [4, 5, 6, 7, 8, 9, 10, 11, 12, 13, 14, 15, 16, 17]. We extracted the following information: demographic data (age, sex), phase of CML when MS emerged, site of MS, treatment modality, response of MS, and overall clinical outcome (Table 2).

**TABLE 2:** Published case reports on radiotherapy for MS in CML.

Study (year)	Age/sex	Phase of CML	Site of MS	MS after initial diagnosis	Treatment	Treatment response	Overall survival (OS) after MS diagnosis
Ali et al. (2022) [8]	26/M	Chronic	Left maxilla	3 years	Imatinib 400 mg/d before MS; dasatinib 140 mg/d; chemotherapy, and followed by RT.	Reduction in the left maxillary mass; awaiting BMT	NR
Nanote et al. (2022) [13]	69/F	Chronic	Right parotid gland	3 years	Imatinib 400 mg bd and later, dasatinib 50 mg bd	NR	NR
Hu et al. (2021) [17]	40/M	Chronic	Left upper arm, left anterior thigh and right posterior thigh	At diagnosis	Cytarabine followed by imatinib later	Resolution of thigh nodules and reduction of arm nodules; BC at 4 months	NR
Han et al. (2020) [12]	37/M	Chronic	Spinal cord (Epidural mass T2 to L2 level)	1 year	Surgery and imatinib; later switched to dasatinib	CR	Alive and in remission at 1‐year follow‐up
Lee et al. (2020) [15]	16/M	Chronic	Right thigh	At diagnosis	Imatinib	CHR at 3 months; follow‐up MRI and PET 6 months showed a healed scar	NR
Kabadi et al. (2017) [16]	72/M	Chronic	Intracranial	At diagnosis	Surgery followed by imatinib 400 mg/d	CR	Alive and well at the time of the report
Dassapa et al. (2017) [4]	35/M	Chronic	Right leg	3 years	Imatinib 400 mg/d; later increased to 600 mg/d	CHR at 3 months; BP at 10 months	11 months
	39/F	Chronic	Right gluteal region	At diagnosis	Imatinib 600 mg/d	CHR at 3 months	16 months
	47/M	Chronic	Right gluteal region	8 months	Imatinib 400 mg/d; later changed to nilotinib 800 mg/d	CHR at 3 months	36 months
	54/F	Chronic	Left thigh	At diagnosis	Imatinib 600 mg/d; later increased to 800 mg/d	No CHR; AP at 8 months; BC at 10 months	12 months
	36/M	Chronic	Left shoulder, cervical lymph node	3 months	Imatinib 400 mg/d; later increased to 800 mg/d	No CHR; BC at 8 months	9 months
	47/M	Chronic	Upper back	2 years	Imatinib 400 mg/d; later increased to 800 mg/d	CHR at 3 months	28 months
	54/F	Chronic	Right leg	11 months	Imatinib 400 mg/d; later increased to 600 mg/d	CHR at 2 months	18 months
	38/M	Chronic	Right inguinal lymph node	6 months	Imatinib 400 mg/d; later increased to 800 mg/d	No CHR: BC at 2 months	3 months
Vasconcelos et al. (2017) [5]	42/F	Chronic	Cutaneous nodules on trunk and limbs	At diagnosis	Imatinib 600 mg/d	CHR	NR
Kittai et al. (2014) [14]	30/M	Chronic	T6 vertebra and paraspinal soft tissue	42 months	Surgery; AML‐type chemotherapy + ponatinib+ haploidential allogeneic BMT	CR	Alive/In remission (1+ year post‐transplant)
Kumar et al. (2013) [6]	25/F	Chronic	Cutaneous nodules on the right and left thigh	At diagnosis	Imatinib	CHR	Followed up for 3 and a half years; doing well
Paydas et al. (2006) [7]	59/M	Chronic	Humerus	23 months	Amputation	NR	23 months
	20/F	Chronic	Abdominal mass	At diagnosis	HU	NR	0.3 months
	55/F	Chronic	Soft tissue/lymph node	At diagnosis	HU	NR	19 months
	26/F	Chronic	Pelvic bone	12 months	Imatinib	Progress to BC	12 months
	16/F	Chronic	Lymph nodes	2 months	RT	NR	96 months
	70/M	Chronic	Pericardial tamponade	5 months	Pericardial drainage	NR	0.5 months
Cozzi et al. (2004) [9]	39/M	Chronic	Left humerus	13 years	High‐dose imatinib + chemotherapy + RT	Progressive local disease	Died of progressive local disease and infection 8 months later
Mahendra et al. (1994) [10]	48/M	Chronic	Extradural with cauda equina syndrome	6 months	HU and interferon before; AML‐type chemotherapy followed by RT	Progressed to BC	Died 4 months later
Gittin et al. (1989) [11]	41/M	Chronic	Left axillary lymph nodes	At diagnosis	RT and HU followed by chemotherapy during relapse	Resolved but relapsed 6 months later and progressed to BC	Died 14 months later with sepsis
	49/M	Chronic	Left femur	10 months	Busulfan before MS; RT for first MS followed by surgery during recurrence	Resolved MS with RT but recurred 5 months later; progressed to BC at 1 month later	Died due to infection

Abbreviations: AML, acute myeloid leukaemia; BC, blast crisis; BMT, bone marrow transplantation; CHR, complete haematological response; CR, complete response; F, female; HU, hydroxyurea; MRI, magnetic resonance imaging; MMR, major molecular response; M, male; MS, myeloid sarcoma; NR, not reported; PET, positron emission tomography; PD, progressive disease; RT, radiotherapy.

For these 27 patients with MS and CML‐CP, the male‐to‐female ratio was 1.7:1, and the median age was 40 years (ranging from 16 to 72 years). Most of the MS (*n* = 17) were diagnosed months after the diagnosis of CML‐CP, and the remaining 10 had MS diagnosed at the same time as CML‐CP. MS affected various sites, including the musculoskeletal system (18 cases), lymph node (3 cases), gastrointestinal system (2 cases), heart (1 case), spinal cord (2 cases), and brain (1 case) [4, 5, 6, 7, 8, 9, 10, 11, 12, 13, 14, 15, 16, 17].

With regards to the treatment strategy, 20 of these 27 patients received TKI alone [4, 5, 6, 7, 13, 15]. Eleven of them responded well to imatinib with an OS ranging from 9 months to 36 months. Dassapa et al. showed eight cases of MS in CML‐CP responding to higher doses of imatinib (600 mg to 800 mg daily), with OS ranging from 3 months to 36 months. Unfortunately, two patients did not achieve complete haematological response (CHR) and transformed to blast crisis in less than a year. Another patient achieved CHR at 3 months but transformed to blast crisis at 10 months [4].

Four patients, including ours, had systemic chemotherapy, imatinib, and radiotherapy at various time points [8, 9]. Kittai et al. demonstrated a case with MS of the spine that achieved remission after surgical intervention, systemic chemotherapy, TKI, and allogeneic stem cell transplant. Cozzi et al. reported a case of aggressive MS 13 years after the diagnosis of CML‐CP, which failed to respond to all treatments, including high‐dose imatinib. The patient eventually died of progressive local disease and mycotic pulmonary infection 8 months later [9]. A patient reported by Ali et al., on the other hand, had a significant reduction of the left maxillary mass and was planned for allogeneic stem cell transplantation. However, the survival outcome was not reported [8]. As for our patient, the uterine MS did not respond to systemic chemotherapy and imatinib at first, but she achieved complete resolution of the MS after a course of radiotherapy. She also achieved and maintained deep molecular response (MR^4.5^) over the past decade with imatinib.

Han S et al. and Kabadi K. et al. reported two cases that responded well to combination treatment with surgery and TKI [12, 16]. Paydas et al. reported a patient with MS of the humerus 23 months after the diagnosis of CML‐CP. The subject underwent amputation only, and he survived for up to 23 months [7].

Prior to 1998, before imatinib was available, Mahendra et al. reported a CML‐CP which was initially treated with hydroxyurea and later, interferon. Then MS occurred 6 months later in the spinal cord, causing cauda equina syndrome, and this was treated with systemic chemotherapy and radiotherapy. However, the disease progressed to blast crisis, and the patient succumbed 4 months later [10]. Gittin et al. reported 2 CML patients with MS who initially responded well to RT (one combined with hydroxyurea and chemotherapy; the other with Busulfan), but both eventually succumbed to blast crisis [11].

## 5 Discussion

Given the limited evidence, a definitive recommendation for managing MS in CML‐CP remains difficult. According to the European Leukaemia Net (ELN) definition, any CML patient with extramedullary blast proliferation (i.e., MS), regardless of the phase of CML, is considered to be in blast crisis [18]. In other words, MS in CML‐CP should be treated as CML in blast crisis (CML‐BC) and should ideally receive AML‐type intensive chemotherapy, TKI, and allogeneic HSCT in transplant‐eligible patients. The European Group for Blood and Marrow Transplantation (EMBT) recommended allogeneic HSCT for CML‐BC after debulking with a combination of induction chemotherapy and second‐generation, third‐generation, or fourth‐generation TKI. EBMT also introduced a reduced‐intensity conditioning regimen (RIC) for older patients and patients with comorbidities. However, this was noted to carry a high risk of relapse as compared to myeloablative conditioning. Though HSCT is the curative treatment for the advanced phase of CML, it is associated with considerable morbidity and mortality. The advent of TKI since 1998 has improved the 5‐year OS tremendously. The outcomes of allogeneic stem cell transplantation in CML, on the other hand, are often still not satisfactory, with long‐term survival rates below 20%, as per a large EBMT analysis [19]. Additionally, the availability of HLA‐compatible donors, the cost of transplantation, transplant‐related morbidity and mortality pose significant challenges for both clinicians and patients.

Our patient did not respond to the combination of AML‐type chemotherapy and imatinib initially. However, she eventually achieved complete resolution of the pelvic MS after a course of local radiotherapy. As for her CML, she responded well to imatinib and achieved sustained deep molecular response for the past 10 years. Hence, despite the known poor outcome for CML‐BC, our patient achieved excellent results with a combination of TKI and radiotherapy without going through allogeneic HSCT. This is the first reported case of MS in CML‐CP achieving 10 years of long‐term treatment remission with a combination of radiotherapy and TKI. The long‐term TKI therapy has presumably suppressed the BCR‐ABL clones, inducing a sustained major molecular response up to 10 years, while radiotherapy exerted good local control of the MS, as MS is often radiosensitive [20, 21, 22]. This highlights the feasibility of this treatment strategy for MS in CML‐CP, especially in the cohort of patients who may not be able to tolerate intensive chemotherapy and allogeneic HSCT. We postulate that more potent second and third‐generation TKIs may result in better outcomes. Given the rarity of this clinical entity, further studies are crucial in optimizing the treatment strategy of MS in CML‐CP. The current review's findings are inherently limited by publication bias, heterogeneity in reporting spanning several decades, and reliance on small sample sizes, which prevents definitive, evidence‐based recommendations for this rare presentation.

## 6 Patient's Perspective

“When I first learned I had blood cancer, I was very worried and scared. But with the treatment and care from my doctors, my condition slowly improved. Now, after 10 years, I am still doing well and able to live a normal life. I am very thankful for the care and support I received.”

## Author Contributions

**Kar Ying Yong:** conceptualization, investigation, funding acquisition, methodology, validation, visualization, writing – original draft, writing – review and editing, supervision. **Soo How Lim:** conceptualization, funding acquisition, methodology, validation, visualization, investigation, writing – original draft, writing – review and editing. **Lee Gong Lau:** writing – original draft, writing – review and editing, supervision. **Lee Ping Chew:** supervision, writing – original draft, writing – review and editing.

## Funding

The authors have nothing to report.

## Ethics Statement

This case report is registered with the Malaysian National Medical Research Register (NMRR ID‐24‐02221‐VVM) and is exempt from ethical review as this case report is strictly observational.

## Consent

The patient gave consent for the results to be published. When the results of the case report study are published, no personal information will be revealed, and the patient will not be identified.

## Conflicts of Interest

The authors declare no conflicts of interest.

## Data Availability

The data that support the findings of this study are available from the corresponding author upon reasonable request.
